# Exploring barriers and educational needs in implementing dual-task training for Parkinson’s disease: insights from professionals

**DOI:** 10.3389/fmed.2024.1325978

**Published:** 2024-04-05

**Authors:** Josefa Domingos, John Dean, Júlio Belo Fernandes, Carlos Família, Sónia Fernandes, Catarina Godinho

**Affiliations:** ^1^Radboud University Medical Centre, Donders Institute for Brain, Cognition and Behaviour, Department of Neurology, Center of Expertise for Parkinson and Movement Disorders, Nijmegen, Netherlands; ^2^Egas Moniz Center for Interdisciplinary Research (CiiEM), Egas Moniz School of Health & Science, Almada, Portugal; ^3^Triad Health, Aurora, CO, United States; ^4^Nurs* Lab, Almada, Portugal; ^5^Molecular Pathology and Forensic Biochemistry Laboratory (MPFBL), Caparica, Portugal

**Keywords:** Parkinson’s disease, dual task training, exercise, barriers, educational needs, professionals, health care, public health

## Abstract

**Introduction:**

There is growing evidence suggesting that dual-task training benefits people with Parkinson’s disease (PD) on both physical and cognitive outcomes. However, there is no known data regarding professionals’ educational needs and barriers to its implementation. This study aimed to explore the barriers and educational needs of healthcare and exercise professionals to integrate dual-task training into their practice with people with PD.

**Methods:**

We conducted a study based on a web survey. Social media channels were used to recruit a convenience sample of exercise and healthcare professionals working with people with PD.

**Results:**

Of the 185 eligible responses, the majority were physiotherapists (68.1%) followed by occupational therapists (10.8%). Most participants attended Parkinson specific training (88.6%) and employed the treatments set up in individual one on-one sessions (58.9%). We identified several barriers to dual-task training implementation, with lack of time (to prepare materials), staying creative and/ or accessing new ideas, unreliable tools for measuring gains, and insufficient expertise as the most referred by participants. The educational needs most referred included accessing examples of interventions in general, knowing what strategies to apply and their application for people with different symptoms.

**Discussion:**

Our results highlight that professionals remain challenged to integrate dualtask training into PD clinical care mainly due to knowledge gaps, difficulties in accessing new ideas, and lack of time.

## 1 Introduction

Parkinson’s disease (PD) is a neurodegenerative disorder characterized by motor symptoms such as tremors, rigidity, bradykinesia, and postural instability, as well as non-motor symptoms including cognitive impairment, depression, and sleep disturbances (1). The prevalence of PD is increasing globally, making it a significant public health concern. According to recent estimates, PD affects approximately 1% of individuals over the age of 60, with the incidence rising with age (2). Alarmingly, it is noted as one of the fastest-growing neurological diseases, with an estimated 10 million people worldwide living with PD (3). This trend underscores the urgent need for enhanced awareness, research, and interventions to address the multifaceted challenges posed by PD. As the global population continues to age, the burden of PD is expected to escalate, emphasizing the importance of proactive measures to improve diagnosis, treatment, and support for individuals affected by this debilitating condition (4).

Pharmacological treatment for PD has proven beneficial, but patients still experience motor and non-motor symptoms that pose challenges for both patients and health professionals (1, 5).

There is growing evidence suggesting that non-pharmacological interventions, such as exercise/physiotherapy (6–9) and cognitive training (7, 10, 11) provides benefit for people with PD on both physical and cognitive outcomes.

Combining interventions at both these levels through the use of dual or multitasking exercises may thus provide a useful new approach for the treatment of older adults (12–14) and PD patients (15–18).

Dual or multitasking refers to effectively handle multiple tasks concurrently during transfers, ambulation, and other gait-related activities (19). It involves engaging in two attention-demanding tasks with distinct objectives, necessitating individuals to allocate attention between tasks or distribute equal attention to both tasks (20, 21). The rationale behind dual-task training lies in its potential to concurrently improve motor and cognitive functions, addressing PD’s multifaceted nature.

Previous studies have demonstrated that dual-task training (DTT) can improve executive function, gait, and balance and reduce the risk of falls and overall functional capacity in older adults (13, 14, 22) and people with PD (23–28).

DTT is becoming increasingly popular for individuals with PD, especially in managing difficulties with gait and balance (8, 24). With the proliferation of such practice, experiential expertise must be complemented by an effective educational component to be truly effective in bridging any gaps along the continuum of care. Hence, data regarding professionals´ educational needs and barriers to its implementation are critical. Here we explore the barriers and educational needs of healthcare and exercise professionals to integrate DTT into their practice with people with PD.

## 2 Methods

### 2.1 Design

This was a quantitative study conducted based on a web-survey.

### 2.2 Sampling and recruitment

We used convenience sampling to recruit healthcare and exercise professionals that integrate DTT into their practice with people with PD. Social media channels such as Instagram, WhatsApp, and professional Facebook groups were utilized to distribute study invitations containing survey access links.

The sample of participants was selected based on the following criteria:

- Be healthcare or exercise professional.
- Previous experience working with people with PD.
- Integrate some form of DTT into their practice with people with PD.
- Willingness to participate in the study.

We excluded participants if they were unwilling to provide informed consent to participate in the study.

### 2.3 Data collection

The survey was conducted using a structured assessment questionnaire, specifically developed for the study. It was developed based on a literature review and with the input of professional healthcare experts. It was hosted on Google™ Forms website.

The survey had three pages: one with study information, another with questions about participant sociodemographic status and professional background, and a final page to explore barriers and educational needs when providing DTT to people with PD. A pre-test was conducted with 10 participants. They were briefed on the purpose of the survey and the wording of the introduction and questions. This feedback allowed researchers to conclude that the survey was objective, comprehensive, and there were no questions that could be ambiguous or equivocal.

It was estimated that the survey would take around 8 min to complete. The survey period was May 1st, 2021, until August 30th, 2021.

### 2.4 Data analysis

The statistical analysis of the questionnaires was performed using the R language and environment for statistical computing v. 4.1.2 (29), with RStudio as integrated development environment v.2021.09.0 (30). Descriptive statistic measures of count, mean, standard deviation, median, minimum, maximum, and range were computed for sample characterization, using the function table1 from the table1 v.1.4.2 library for R (31). Any incomplete questionnaires were excluded from the analysis.

### 2.5 Ethical considerations

This study complies with principles of the Declaration of Helsinki. The study protocol was analyzed and approved by the Egas Moniz Research Ethics Board (Date: April 2021 ID: 964).

The survey was set up in a way that participant had to answered “YES” or “No” indicating that he/she has read the consent information and agrees to participate. Only the participants who answered “YES” to the informed consent question were directed to the research survey. Participants who answered “NO” to the informed consent question were directed to the end of the survey. Participants were free to decide not to answer any question, change or review their responses, or voluntarily quit at any time. Data was conducted in compliance with ethical principles guaranteeing the participants’ anonymity and confidentiality. Therefore, all data was free of any personally identifying information, including IP addresses or other electronic identifiers.

Data files are stored on a password protected computer in a restricted access locked file cabinet at Egas Moniz University.

## 3 Results

### 3.1 Characteristics of the participants

From the 185 eligible responses (Table 1), most participants were physiotherapist (68.1%), followed by occupational therapists (10.8%). They perform their practice in America (83.8%), followed by Europe (12.4%) and are graduate (44.9%). Regarding the participants professional experience, the mean of years of practice was 18.8 ± 11.1 (range 1–44 years) and the mean of years of practice with people with PD was 12.5 ± 9.05 (range 0.5–40 years). Most participants had attended Parkinson specific training (88.6%) and provided one on one sessions (58.9%), and group sessions (37.3%).

**Table 1:** Characteristics of the participants.

	Overall (*N* = 185)
Age (years)	
Mean (SD)	45.6 (11.1)
Median [Min, Max]	46.0 [23.0, 73.0]
Continent	
America	155 (83.8%)
Asia	2 (1.1%)
Australia	5 (2.7%)
Europe	23 (12.4%)
Discipline	
Athletic trainer and related	13 (7.0%)
Exercise physiology	7 (3.8%)
Fitness	2 (1.1%)
Medicine	2 (1.1%)
Neurology	2 (1.1%)
Occupational therapy	20 (10.8%)
Physiotherapy	126 (68.1%)
Speech language pathology	13 (7.0%)
Education	
Undergraduate	2 (1.1%)
Graduate	83 (44.9%)
Master	71 (38.4%)
Doctor	29 (15.7%)
Years of Practice	
Mean (SD)	18.8 (11.1)
Median [Min, Max]	18.0 [1.00, 44.0]
Years of practice with Parkinson’s disease patients	
Mean (SD)	12.5 (9.05)
Median [Min, Max]	10.0 [0.500, 40.0]
Parkinson specific training	
No	21 (11.4%)
Yes	164 (88.6)
Treatments setup	
Individual one on one	109 (58.9%)
Group setting	7 (3.8%)
Both	69 (37.3%)

### 3.2 Integration of dual task training

Participants integrated DTT in their practice very frequently (37%) or frequently (37.5%), mostly in individual sessions (88.1%) (Table 2). This type of training was primarily applied to people with PD in early stages (89.7%), and people with mild cognitive impairment (78.9%) (Table 3).

**Table 2:** Frequency of applying dual-task training.

	Overall (*N* = 185)
Rarely	2 (1.0%)
Occasionally	28 (13.5%)
Frequently	78 (37.5%)
Very Frequently	77 (37.0%)
Missing	23 (11.1%)

**Table 3:** Clinical situations in which dual-task training is used in practice.

	Overall (*N* = 185)
People in early stages	166 (89.7%)
People with self-perceive cognitive difficulties	136 (73.5%)
People with mild cognitive impairment	146 (78.9%)
Individual sessions	163 (88.1%)
Group treatments	75 (40.5%)

### 3.3 Educational needs to integrate dual task training

Participants identified 21 educational needs that impact their ability to integrate DTT in their practice (Table 4). Overall, participants identified the need for strategies to apply in DTT, how to structure DTT sessions, duration, and measurement tools to assess patients’ status before, during, and after the training program.

**Table 4:** Educational needs regarding dual task training.

	Overall (*N* = 185)
Research and evidence behind its use	112 (60.5%)
Examples of interventions in general	127 (68.6%)
What DTT strategies improve balance	111 (60.0%)
What DTT strategies improve gait	110 (59.5%)
What DTT strategies improve transfers	79 (42.7%)
What is the right time to use DTT with people with PD	103 (55.7%)
What DTT strategies can be used in the late stages	111 (60.0%)
What is the optimal frequency and duration of such treatments or exercises	103 (55.7%)
Which dual task measurement tool(s) should be used during history taking	67 (36.2%)
Which dual task measurement tool(s) should be used during physical examination	86 (46.5%)
When should dual task evaluation and reassessments be carried out	56 (30.3%)
Safety issues and emergencies when using DTT	63 (34.1%)
Inclusion and exclusion criteria to whom to apply it too	67 (36.2%)
Its application to people with freezing of gait	100 (54.1%)
Its application to people with cognitive impairment	109 (58.9%)
Its application to people with atypical Parkinsonism	91 (49.2%)
How to integrate it safely in group settings	72 (38.9%)
Recommendations for equipment and environment adaptations	55 (29.7%)
What Parkinson-specific expertise is required for professionals to do DTT	55 (29.7%)
What information should be provided to patients, and how to discuss expectations about this type of training with patients	65 (35.1%)
How to provide training to other professionals regarding DTT	54 (29.2%)

DTT, Dual task training; PD, Parkinson’s disease.

The majority of participants (68.6%) stated that they needed examples of DTT interventions. A primary educational need for any evidence-based practice professional is to identify research regarding intervention and evidence behind its use. In this study, participants (60.5%) acknowledged this need. Additionally, participants expressed the need for education on the appropriate timing of DTT use for individuals with PD (55.7%), the implementation of strategies to achieve rehabilitation goals, including balance improvement (60.0%), gait improvement (59.5%), and transfer improvement (42.7%). Furthermore, they emphasized the importance of determining the optimal frequency and duration of treatments (55.7%), as well as the usage of measurement tools to evaluate patients’ conditions before and during the training program.

Considering that participants drive from different fields of knowledge, it is vital to acquire knowledge and skills to PD. This educational need to have Parkinson-specific expertise to do DTT was felted by 29.7% of participants.

Another identified educational need was defining inclusion and exclusion criteria to whom to apply it (36.2%) and its application to people with different health statuses/ symptoms (freezing of gait, 54.1%; cognitive impairment, 58.9%; atypical Parkinsonism 49.2%, and PD late stages 60%).

Managing the patients’ expectations and providing information regarding DTT (35.1%), maintaining patients’ safety during DTT (34.1%) to integrate this type of training safely in group settings (38.9%), and recommendations of equipment and environment adaptations (29.7%) to provide DTT were also educational needs identified by participants.

Finally, 29.2% of the participants also recognize the importance of providing education to other professionals about DTT.

### 3.4 Barriers to integrate dual task training

Participants identified 11 barriers to integrate DTT in their practice (Table 5). The lack of strong scientific evidence (13.5%) and reliable tools for measuring gains (34.1%) were barriers reported by participants to integrate DTT into their practice. Another challenge highlighted by participants was a lack of expertise (25.9%) required to apply DTT. Importantly, we also see participants reporting that the lack of appropriate caseload (19.5%) and physical resources (15.7%) also barriers that can significantly impact the professionals’ ability to perform DTT as patient-professionals ratios are linked to the quality of care.

**Table 5:** Barriers to integrate dual task training in practice.

	Overall (*N* = 185)
Insufficient expertise	48 (25.9%)
Lack of robust scientific evidence to support its use	25 (13.5%)
Lack of appropriate caseload	36 (19.5%)
Lack of physical resources	29 (15.7%)
Lack of financial resources	18 (9.7%)
Lack of time in general	56 (30.3%)
Lack of time to prepare materials, exercises, and new ideas	69 (37.3%)
Being able to stay creative and/or accessing new ideas	95 (51.4%)
Resistance from patients	26 (14.1%)
Lack of reliable tools for measuring gains	63 (34.1%)
Safety	7 (3.8%)
None	4 (2.2%)

Other barriers identified by participants were the lack of time in general (30.3%), the lack of time to prepare materials and exercises (37.3%), and being able to stay creative and/or access new ideas (51.4%).

The lack of financial resources (9.7%) is a barrier that can impact the healthcare and exercise professionals’ ability to perform DTT because the economic burden or insurance coverage can limit the time and number of training sessions for each patient.

Finally, participants reported patient resistance (14.1%) and safety concerns (3.8%) as barriers to implementing DTT. They also expressed concern about the risk of falls during group sessions with less supervision due to lower client-to-healthcare/exercise professional ratios.

## 4 Discussion

Using the DTT paradigm to enhance healthcare outcomes in individuals with PD is an emerging area of interest acknowledged in international guidelines (8). Moreover, recent studies underscore the effectiveness of DTT in people with PD (16–18, 32). As a result, health and exercise professionals must acquire comprehensive knowledge about this type of training and develop the necessary skills to implement DTT safely and effectively. It is crucial to identify the barriers and educational needs experienced by healthcare and exercise professionals to integrate DTT into their practice with people with PD.

In this study, most participants expressed interest in receiving further training regarding DTT (Table 4). This signifies their awareness of existing knowledge gap. Furthermore, it may also potentially reflect their perception of the importance of DTT in rehabilitating people with PD, suggesting their readiness acquire expertise and develop skills in this area.

Participants identified insufficient expertise as a barrier to integrating DTT in their practice (Table 5). This data may be due to the different levels of expertise and knowledge among participants and the awareness of the need for ongoing development of their skills throughout their professional careers to match the changing complexity of healthcare needs (33). It also highlights the need to develop specific educational programs regarding DTT training that may continuously incorporate the growing evidence of DTT.

Designing a comprehensive DTT program involves several pivotal components. This encompasses evaluating the suitability of individuals for the training, carefully selecting treatment strategies aligned with desired outcomes, establishing the optimal frequency and duration of sessions, and identifying requisite equipment and environmental adaptations (8, 34). In this study, participants recognized these components as pivotal educational needs (Table 4). Timely attention to these areas is imperative, as they significantly shape the capability of healthcare and exercise professionals to effectively and safely implement DTT. This underscores the urgency for dedicated research to address these inquiries. Prioritizing DTT research within healthcare is paramount, as it underpins the delivery of optimal care, ultimately aiming to achieve better patient outcomes (35).

Research on best measurement tools is also critical as participants also identified the need-to-know which measurement tools to use to assess patients’ status (Table 4). Outcome measures are essential tools for guiding clinical decision-making. By using measurement tools, healthcare, and exercise professionals will have access to data that allows them to make decisions about how to best care for patients. It will also help professionals predict who might benefit most from a particular intervention and identify patients’ improvements after applying it (36, 37). Using measurement tools to assess patients’ status is a correct practice and a crucial feature for improving clinical care (38).

Working with patients with chronic conditions can be difficult due to several reasons. To keep the rehabilitation programs challenging and engaging, healthcare and exercise professionals must design new exercises, constantly inventing or adapting older ones. In our study we identified that professionals struggled with insufficient time in general, the lack of time to prepare materials and exercises, and staying creative and/or accessing new ideas (Table 5). We perceive these barriers as interlinked, as time emerges as a key factor that impacts the ability of healthcare and exercise professionals to adequately plan, structure, and implement DTT sessions for individuals with PD. This aligns with findings from other studies that reported that professionals struggle to continue developing new exercises so that training sessions are challenging and engaging for people with PD (15, 39). This can also be relevant for the patients’ adherence as previous studies reported that keeping the exercise programs challenging and engaging can influence the patient’s motivation and compliance to perform ongoing DTT exercise (39–41). Educational courses, peer exchanges, and participation in social media groups can serve as valuable tools in mitigating these challenges (42, 43).

Furthermore, managing the patients’ expectations and providing information regarding DTT is an educational need that can significantly impact the persons’ health outcomes’ (44, 45). By managing patients’ expectations, healthcare and exercise professionals can help them adjust their aims, enhance their healthcare experience, anticipate concerns, and prevent them from arising, increasing patients’ confidence in the care provided (44, 45).

Regarding patient’s safety concerns during DTT (Table 5), in our view it is a pertinent and vital education need. We know that when a person is required to perform DTT, the attention is divided between tasks, resulting in the lack of sufficient attentional resources due to a decrement in one or both concurrent tasks leading to an increased risk of losing balance and falling (46, 47). Therefore, during its implementation, it should be recognized that DTT may involve an increased risk of accidents, particularly in group sessions. Maintaining a safe environment should be a priority for all healthcare and exercise professionals. Healthcare and exercise professionals can employ various strategies to reduce or mitigate these safety risks. Selecting appropriate exercise venues, identifying suitable forms of assistance, and determining optimal exercise modalities are key factors in enhancing patient safety. Utilizing safety harnesses, maintaining proximity to the patient, implementing a slower progression of added tasks, providing clear activity instructions, and using cueing strategies to ensure optimal performance can also be very helpful. To minimize risk in group settings, it is advisable to follow the physiotherapy guideline for Parkinson’s disease (8), which recommends a maximum ratio of eight participants per therapist. In such situations, the risk should be assessed, and more than one professional’s need for session supervision should be considered.

Expertise plays a pivotal role in ensuring safety; the level of expertise will inevitably shape the clinical judgment of healthcare and exercise professionals, as well as the quality of training delivered. The greater the knowledge possessed by the professional, the higher the level of safety that can be upheld. Thus, possessing ample expertise in DTT is a central factor in ensuring high-quality patient care. This can be achieved through training courses and also accessing more people with PD (8).

Overall, we identified several barriers (Table 5) and educational needs (Table 4) that must be addressed to disseminate DTT as an effective and safe care offer for all people with PD. Based on the outcomes of our study, providing healthcare and exercise professionals with comprehensive training covering a variety of programmatic content is essential for the effective implementation of DTT in patients with PD.

In addition to developing the necessary skills to become experts, the results underscore the critical importance of allocating time in the work plan of healthcare and exercise professionals to allow for session planning, aiming to avoid excessive exercise repetition and promote better adherence of individuals with PD to DTT.

At an institutional level, healthcare systems that offer this type of treatment are encouraged to increase the availability of community programs that allow individuals to benefit from these interventions (4).

From a financial perspective, it is suggested that evidence-based practices, including DTT, should be covered as a health insurance benefit. This ensures that DTT is recognized as a viable treatment option and included in the insurance coverage (4, 48).

### 4.1 Study strengths and limitations

This study is one of the pioneering efforts in the field due to the scarcity of research addressing healthcare and exercise professionals’ barriers and educational needs concerning DTT for individuals with PD. By delving into this area, the study offers valuable insights into the challenges professionals encounter as they integrate DTT into their practice.

Web-based surveys conducted rigorously can provide faster and more cost-effectively evidence than traditional approaches (49), but have limitations. One of these limitations is the researchers’ inability to explore open-ended responses with immediate follow-up questions, which would allow for a deeper understanding of the subject matter. In addition, as noted in previous studies that relied on data collected through online surveys, selective participation can be a specific limitation due to the automatic exclusion of potential participants who do not use social media platforms. Another limitation arises from convenience sampling, which may introduce selection bias as participants were recruited through social media and professional groups. Consequently, this method may not capture the perspectives of less active professionals in online communities.

While the study offers valuable insights into integrating DTT in PD care, researchers and practitioners should consider these strengths and limitations when interpreting and applying the findings to clinical practice and future research endeavors.

## 5 Conclusion

This study explores the barriers and educational needs of healthcare and exercise professionals integrating DTT into their practice with people with PD. Our results highlight that professionals remain challenged to integrate DTT into PD clinical care largely due to knowledge gaps, difficulties in accessing new ideas, and lack of time. The participants mostly reported needing examples of interventions and strategies applied to individuals with varying symptoms to address educational needs.

## Data availability statement

The raw data supporting the conclusions of this article will be made available by the authors, without undue reservation.

## Ethics statement

The studies involving humans were approved by Egas Moniz Research Ethics Board. The studies were conducted in accordance with the local legislation and institutional requirements. The participants provided their written informed consent to participate in this study.

## Author contributions

JDo: Conceptualization, Data curation, Formal analysis, Investigation, Methodology, Supervision, Writing – original draft, Writing – review & editing. JDe: Conceptualization, Formal analysis, Investigation, Methodology, Supervision, Writing – original draft, Writing – review & editing. JF: Formal analysis, Methodology, Writing – original draft, Writing – review & editing. CF: Formal analysis, Methodology, Supervision, Writing – original draft, Writing – review & editing. SF: Formal analysis, Methodology, Supervision, Writing – original draft, Writing – review & editing. CG: Conceptualization, Data curation, Formal analysis, Investigation, Methodology, Supervision, Writing – original draft, Writing – review & editing.
