# Supplementary material for: Exploring barriers and educational needs in implementing dual-task training for Parkinson’s disease: insights from professionals
Source: Front Med (Lausanne). 2024 Apr 5;11:1325978. doi: 10.3389/fmed.2024.1325978 (PMC11032016; doi:10.3389/fmed.2024.1325978)
Supplement: Supplementary file 1 [file Table_1.DOCX]

Age: __________

Country: ________________

What is your discipline:

- Physiotherapy
- Occupational therapy
- Speech language pathology
- Athletic trainer and related
- Exercise physiologist
- Other: ________________

What is your level of education or degree? ______________________

Have you received any specific training on Parkinson's disease? Yes/No

How long have you been practicing? ____________________________

How long have you been practicing with people with Parkinson´s? _________________

How do you usually deliver these treatments:

- Individual one on one
- Group setting
- Both
- Other: ______________

Do you currently use dual-task or multitask training in your treatments? Yes/No

How frequently?

- Very Frequently
- Frequently
- Occasionally
- Rarely
- Very rarely
- Never

In what clinical situations do you use it? (select all that apply)

- People with PD in early stages (stage I and II)
- People with self-perceive cognitive difficulties
- People with mild cognitive impairment
- Individual sessions
- Group treatments
- Other: _______________

Please indicate your primary educational needs regarding dual-task training for individuals with Parkinson's disease:

- Research and evidence behind its use
- Examples of interventions in general
- What DTT strategies improve balance
- What DTT strategies improve gait
- What DTT strategies improve transfers
- What is the right time to use DTT with people with PD
- What DTT strategies can be used in the late stages
- What is the optimal frequency and duration of such treatments or exercises
- Which dual task measurement tool(s) should be used during history taking
- Which dual task measurement tool(s) should be used during physical examination
- When should dual task evaluation and reassessments be carried out
- Safety issues and emergencies when using DTT
- Inclusion and exclusion criteria to whom to apply it too
- Its application to people with freezing of gait
- Its application to people with cognitive impairment
- Its application to people with atypical Parkinsonism
- How to integrate it safely in group settings
- Recommendations for equipment and environment adaptations
- What Parkinson-specific expertise is required for professionals to do DTT
- What information should be provided to patients, and how to discuss expectations about this type of training with patients
- How to provide training to other professionals regarding DTT
- Other(s)

If you selected 'Other(s)' in the previous question, please indicate the specific educational needs.

__________________________________________________________________

What are the main barriers you identify in integrating dual-task training for individuals with Parkinson's?

- Insufficient expertise
- Lack of robust scientific evidence to support its use
- Lack of appropriate caseload
- Lack of physical resources
- Lack of financial resources
- Lack of time in general
- Lack of time to prepare materials, exercises, and new ideas
- Being able to stay creative and/or accessing new ideas
- Resistance from patients
- Lack of reliable tools for measuring gains
- Safety
- None
- Other(s)

If you selected 'Other(s)' in the previous question, please indicate the specific barriers you have encountered.
